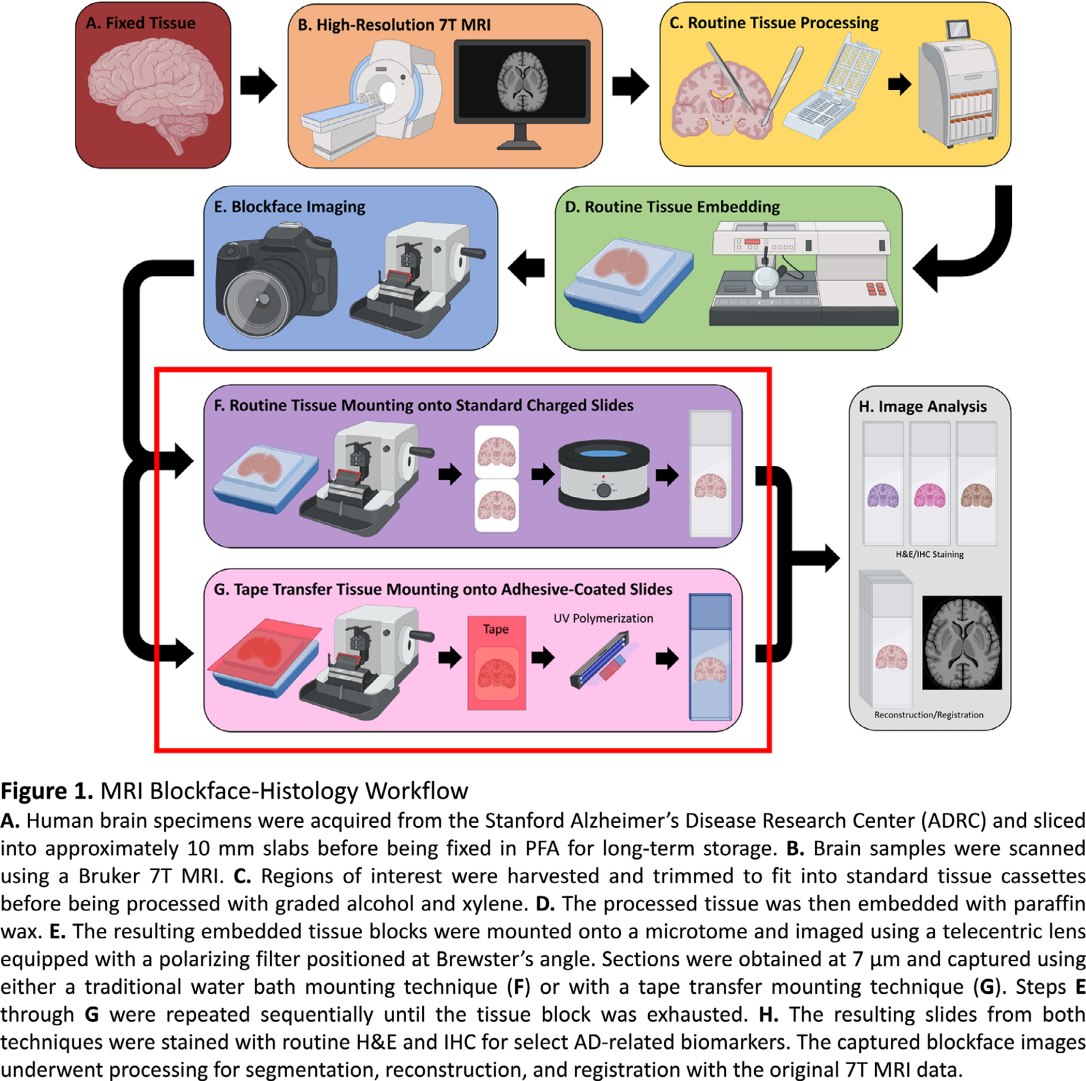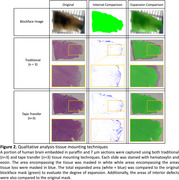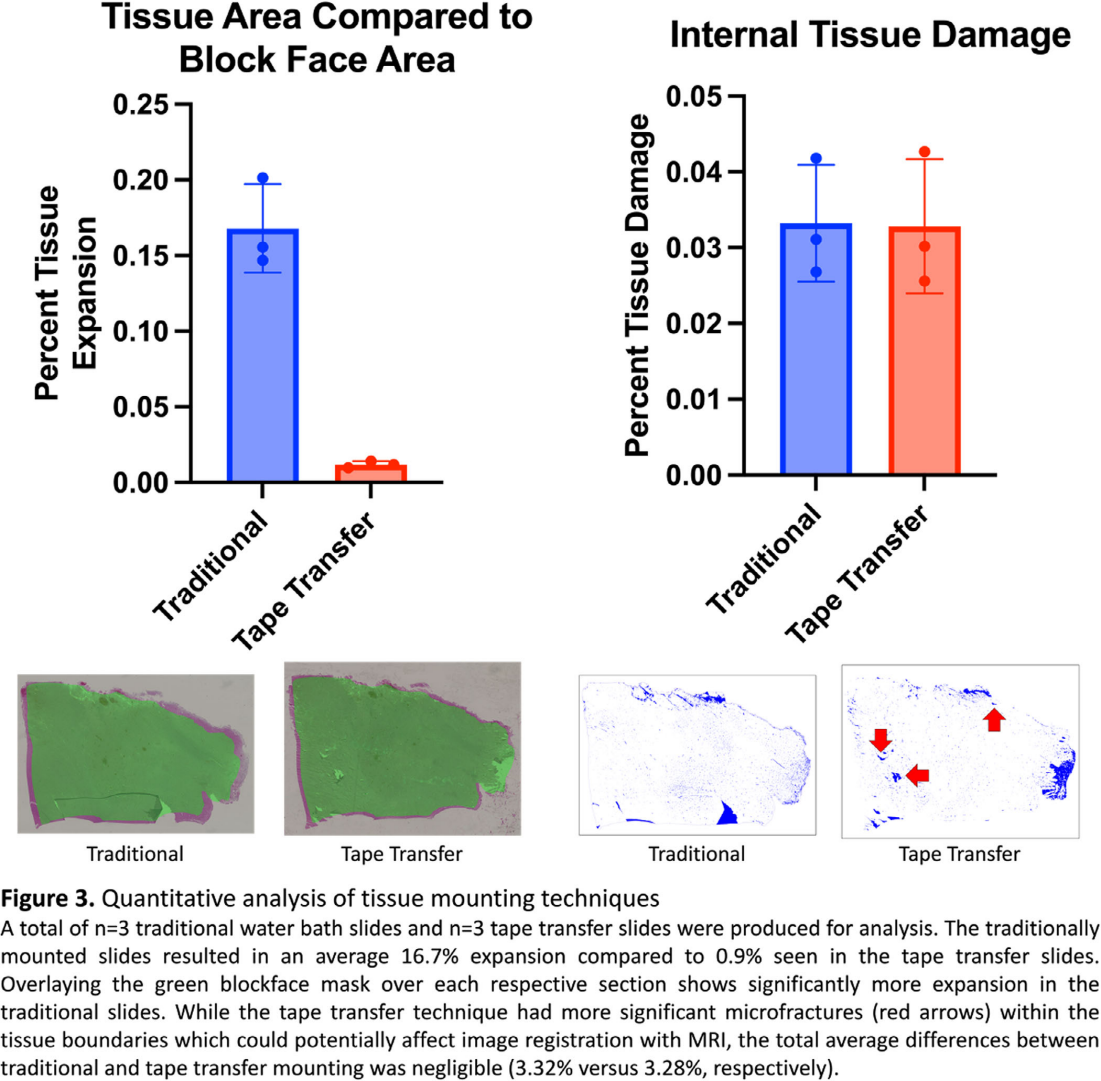# Evaluating quality of routine vs. tape‐transfer histology sections for 3D reconstruction of paraffin embedded brain tissue and correlation with 7T MRI

**DOI:** 10.1002/alz.093158

**Published:** 2025-01-09

**Authors:** William Hai Dang Ho, Yixin Wang, Hossein Moein Taghavi, Phillip DiGiacomo, Jeffrey Nirschl, Pauline Chu, Marios Georgiadis, Michael Zeineh

**Affiliations:** ^1^ Stanford University, Stanford, CA USA; ^2^ Institute for Stem Cell Biology and Regenerative Medicine / Stanford University School of Medicine, Stanford, CA USA; ^3^ Radiological Sciences Laboratory / Stanford University School of Medicine, Stanford, CA USA; ^4^ Pathology Service Center‐Histology / Stanford University, Stanford, CA USA

## Abstract

**Background:**

Specimen analysis is crucial for identifying imaging and neuropathological signatures. Histology is the gold‐standard, but sample preparation and sectioning induce tissue deformations which hinder quantitative analysis or registration of histology to 3D MRI providing a challenge to the development of MRI biomarkers. Overall, we aim to develop a workflow to correlate histology with high‐resolution MRI at a microscopic level (Figure 1), Here, we evaluate a critical step in this process ‐ the section quality from tissue mounting techniques, comparing: A) traditional water bath (Figure 1F), and B) tape transfer (Figure 1G), for the purpose of image segmentation and correlation with high‐resolution MRI.

**Methods:**

Fixed human brain specimens were MRI scanned, dehydrated with graded ethanol, xylene‐cleared, and paraffin‐embedded. Before sectioning, the specimen surface was captured by a brightfield microscope (Figure 2, top left, “Blockface image”). Embedded tissue was sectioned at 7 μm using a Leica 100 microtome and mounted on slides using either A) traditional water bath capture onto standard charged slides (Figure 1F), or B) tape‐transfer capture onto adhesive‐coated slides (Alphametrix) (Figure 1G). Slides were hematoxylin and eosin stained followed by imaging using the same microscope settings as the blockface image, and total stained tissue area was calculated. Area of expansion was determined by change from the blockface image (Figure 2, 3rd column). Internal tissue distortions were calculated by subtracting the tissue section mask from the original blockface mask (Figure 2, 2nd column).

**Results:**

The tissue area measurements show that traditional capture resulted in average expansion of 16.7% relative to the blockface image, whereas tape transfer had 0.9% expansion (Figure 3). Both methods had similar amounts of average total tissue damage (approximately 3%), representing regions with tissue in the blockface image where no tissue was present on the slide. Subjectively, tissue distortion was concentrated at the periphery on traditional capture, whereas there were linear striations within internal structures on tape transfer (red arrows). Tissue yield for tape transfer was subjectively near 100% compared to traditional at approximately 60‐70%.

**Conclusion:**

Tape transfer had a more consistent yield and better edge preservation with blockface image but had more internal microfractures